# Mining for Mitochondria: 68 Mitogenomes for Wrasses and Parrotfishes (F: Labridae) From Off‐Target UCE Data

**DOI:** 10.1002/ece3.73270

**Published:** 2026-03-18

**Authors:** Aditya V. Swami, Lauriane M. Baraf, Peter F. Cowman

**Affiliations:** ^1^ College of Science and Engineering, James Cook University Townsville Queensland Australia; ^2^ Institute for Marine and Antarctic Studies University of Tasmania Hobart Tasmania Australia; ^3^ Biodiversity and Geosciences Program Queensland Museum Tropics Townsville Queensland Australia; ^4^ Center for Tropical Bioinformatics and Molecular Biology James Cook University Townsville Queensland Australia

**Keywords:** bioinformatics, labridae, mitogenome, off‐target reads, reef fish, ultraconserved elements

## Abstract

Labridae (wrasses and parrotfishes) is one of the most ecologically diverse families of reef‐associated fishes but remains underrepresented in mitochondrial genomic resources. The availability of complete mitochondrial genomes is critical for both evolutionary and ecological research, since they are increasingly being used across population genetic, phylogenetic, species identification and eDNA studies. A low‐cost method to increase mitogenomic representation is to leverage off‐target reads produced in target‐capture sequencing (TCS). Here we use a recently published ultraconserved elements (UCE) dataset for Labridae to assemble and annotate off‐target reads to produce complete mitogenomes for 68 species within Labridae, 54 of which are novel to NCBI. These novel complete mitogenomes expand the taxonomic coverage of labrid mitogenomes from less than 5% and 12% to almost 13% and 20% on NCBI's RefSeq and Nucleotide databases, respectively. Partial mitochondrial genes were also recovered for 191 additional species in the family. Mitogenome lengths ranged from 16,320 to 17,288 bp with highly conserved protein‐coding genes, rRNAs and tRNAs. The non‐coding D‐loop region showed the most length variation, ranging from 626 to 1556 bp. Species from the Cirrhilabrinae tribe had the longest mitogenomes (average = 17.2 kbp), while species from the Julidinae tribe displayed the broadest size range (16.3–17.1 kbp), likely due to their higher species richness and representation in the original UCE dataset. The mitogenomic phylogenetic reconstruction was strongly supported and revealed topological discordances in the placement of Cirrhilabrinae when compared to published nuclear phylogenies in Labridae. The newly assembled mitogenomes from our study further highlight the utility of off‐target reads in TCS datasets as a cost‐effective source of genomic material, facilitating broader evolutionary and conservation‐based investigations into the Labridae.

## Graphical abstract

By leveraging off‐target reads from a target‐capture UCE dataset, we assembled and annotated complete mitogenomes for 68 species of wrasses and parrotfishes (Family: Labridae), 54 of which are novel to the NCBI. This increases the taxonomic coverage of reference labrid mitogenomes from less than 5% and 12% to almost 13% and 20% of the family on NCBI's RefSeq and Nucleotide databases. We describe and compare the novel mitogenomes across labrid tribes and reconstruct a mitogenomic phylogeny to explore the mitonuclear discordance in this family.

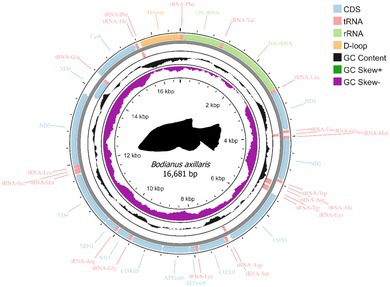

## 1 Introduction

Through selectively enriching and sequencing specific genomic regions, targeted capture sequencing (TCS) has garnered widespread applications across ecological (Jones and Good 2016), evolutionary (Carter et al. 2019; Grover et al. 2012) and biomedical studies (Hagemann et al. 2013; Ng et al. 2009). Recent advances in high‐throughput targeted capture of ultraconserved elements (UCEs) have facilitated the resolution of ancient and recent splits within various invertebrate (Branstetter et al. 2017; Cowman et al. 2020; Faircloth et al. 2015) and vertebrate lineages (Faircloth et al. 2012, 2013; Tea et al. 2022). Off‐target reads from UCE datasets can often yield complete mitochondrial genomes (mitogenomes) as a by‐product (Raposo do Amaral et al. 2015; Smith et al. 2014), providing a cost‐effective alternative to traditional mitogenome capture techniques like shotgun sequencing (Crampton‐Platt et al. 2016; Liu et al. 2016). This method also increases the availability of mitochondrial sequence data on reference databases like NCBI, thereby allowing holistic analyses of nuclear and mitochondrial genomes concurrently at no additional cost (Raposo do Amaral et al. 2015).

Due to their relative availability, mitochondrial genomes have been extensively used across a wide range of studies spanning population genetics (Shao and Barker 2007; Sun et al. 2025), detection of cryptic species (Iannelli et al. 2007), delineation of conservation management units (Feutry et al. 2014) and species identification using barcoding techniques (Antil et al. 2023). More recently, these barcoding techniques have also been applied in eDNA monitoring studies where the presence of threatened species can be detected using a primer made from a 600–800 base pair (bp) fragment of the mitochondrial COX1 gene (Mauvisseau et al. 2020; Vrijenhoek 1994). Additionally, analysing mitogenomes in conjunction with genome‐wide nuclear data has attracted significant interest in molecular phylogenetics, with different datasets often yielding discordant tree topologies across recently diverged lineages and ancestral relationships of rapidly radiating lineages (Alda et al. 2019; Degnan and Rosenberg 2009; Eaton and Ree 2013; Yi et al. 2024). The discordance between nuclear and mitochondrial genetic history is not unexpected given the known variation in rates of evolution between the two genomes (Brown et al. 1979) and the maternal inheritance of the mitochondria (Hutchison III et al. 1974). But the discordance can also be a useful indicator of evolutionary processes such as incomplete lineage sorting, interspecific hybridization and mitochondrial introgression (Klicka et al. 2024; Linnen and Farrell 2007; Wallis et al. 2017), patterns that can often be masked when relying on a single gene nuclear markers. Additionally, the nuclear genome has been shown to have lower rates of mutations when compared to the mitochondrial genomes of most taxa (Allio et al. 2017; Nabholz et al. 2008), with the notable exception of Anthozoa (Hellberg 2006; Shearer et al. 2002). Hence, the mitochondrial genome, either in part or as a whole, has been consistently used in phylogeographic and phylogenetic studies across various invertebrate and vertebrate lineages (Duchene et al. 2012; Inoue et al. 2003; Linard et al. 2018; Morin et al. 2010; Vilstrup et al. 2011), including the very speciose lineage of ray‐finned fish—Actinopterygii, where mitogenomic data have been used to study phylogenetic relationships (Inoue et al. 2003), historical biogeography (Inoue et al. 2009) and molecular evolution (May et al. 2020; Qiu et al. 2014).

**FIGURE 1:**
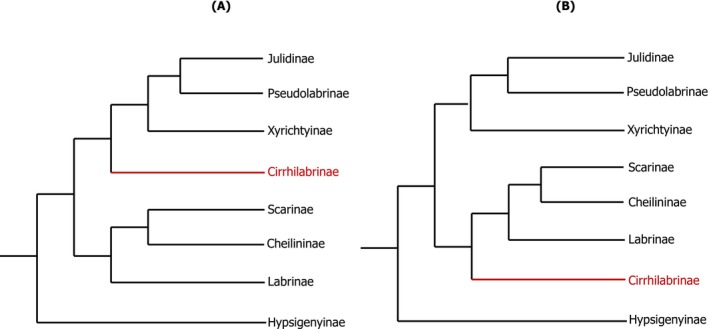
Tree topologies showing the discordant position of tribe (or subfamily) Cirrhilabrinae in the labrid tree of life from (A) a minority of the maximum likelihood phylogenies from Hughes et al. (2023) and (B) the UCE phylogeny from Brownstein et al. (2025) and the majority of maximum likelihood phylogenies in Hughes et al. (2023).

One of the most prominent families within Actinopterygii is Labridae—the wrasses and parrotfishes. Known for their diversity in colouration, size, morphology and behaviour, labrids play a number of essential ecological roles on coral reefs, including but not limited to cleaners, grazers, browsers, bioeroders and corallivores (Clements and Choat 2018). Labrids also span a wide range of geographic distributions, from widespread species like *Bodianus axillaris* (Russell 2010) to narrow‐range endemics such as *Scarus obishime* (Myers et al. 2012). This exceptional diversity of appearance, geographic ranges and function is underpinned by the species richness of Labridae, with 690 valid species across 80 genera and 8 tribes recognized on Eschmeyer's Catalogue of Fishes (Fricke et al. 2026; as of February 2026). Despite the high taxonomic diversity in Labridae, their mitochondrial genetic resources remain relatively underrepresented on the NCBI Reference Sequence (RefSeq) and Nucleotide Databases. While complete mitogenomes for 81 labrid species across 36 genera are available on the Nucleotide database, only 32 species from 19 genera have verified mitogenomes on the RefSeq database at present. Hence, less than 12% and 5% of all species in the family are represented in the Nucleotide and RefSeq databases, respectively. As a result, while studies have been able to analyse several mitochondrial markers to elucidate evolutionary histories and phylogenetic relationships in wrasses (Choat et al. 2012; Cowman and Bellwood 2011; Cowman et al. 2009; Tea et al. 2022; Westneat and Alfaro 2005), no study has conducted a comparative analysis on mitogenomes across this diverse family. Moreover, topological discordances have been shown in tree topologies derived from studies looking at phylogenetic relationships in wrasses, specifically with respect to the placement of the tribe Cirrhilabrinae (Brownstein et al. 2025; Hughes et al. 2023; Figure 1).

As multiple locus nuclear marker datasets are beginning to make way for genome‐scale phylogenetic reconstructions (phylogenomics), particularly in fishes (Alfaro et al. 2018), there is an opportunity to make TCS datasets ‘backwards‐compatible’ with legacy markers by mining off‐target reads for mitogenomes (Baraf et al. 2024) and other markers (Grinblat et al. 2021). Here, we leveraged a recently published target‐capture dataset of UCEs for wrasses and parrotfishes (Brownstein et al. 2025) to assemble, annotate and describe novel mitochondrial genomes from UCE off‐target reads. From all available UCE assemblies in the dataset, our primary goal is to mine, assemble and annotate novel mitochondrial data to increase taxonomic representation of complete mitogenomic data for the family Labridae on NCBI. We also aim to reconstruct a phylogeny for the family using the mitochondrial data produced during this study and existing mitogenomes available on the RefSeq and Nucleotide databases, and additionally analyse any topological discordances between our phylogeny and other published labrid phylogenies. All novel mitogenomes are made available for future studies through NCBI (Bioproject PRJNA1335850).

## 2 Materials and Methods

### 2.1 Mitogenome Assembly, Annotations and Length Comparisons

We analysed a target‐capture UCE dataset from Brownstein et al. 2025 (NCBI GenBank BioProject PRJNA1114898), generated using the Acanthomorph 2.5Kv1 UCE bait set (Alfaro et al. 2018). This dataset comprised 388 Illumina paired‐end reads for 311 distinct species across 68 genera in Labridae, with species ID verified through expert taxonomic assessment (see details in Brownstein et al. 2025). We used fastp (Chen et al. 2018) to assess the quality of demultiplexed paired‐end reads in the dataset and remove adapter sequences.

Prior to assembly, we compiled all complete mitochondrial genomes for the family Labridae from the NCBI RefSeq and Nucleotides databases (Benson et al. 2012; O'Leary et al. 2016), excluding unverified and potentially erroneous mitogenomes, into a reference dataset (see Table S1). Any duplicates (see Table S2) or incomplete mitogenomes—missing any standard vertebrate mitochondrial feature (13 protein‐coding genes [PCGs], 2 ribosomal RNAs [rRNAs], 22 transfer RNAs [tRNAs] or the control region [D‐loop])—were subsequently removed from the dataset. Gene nomenclature was reviewed across the retained mitogenomes, and any inconsistent annotation names were manually standardized (e.g., ‘COI’ changed to ‘COX1’ and ‘control region’ changed to ‘D‐loop’) following Baraf et al. (2024) to ensure consistent downstream annotation of novel mitochondrial data. The curated mitogenomes in the reference dataset (hereafter referred to as ‘reference mitogenomes’) were then concatenated into (i) a single comprehensive multi‐FASTA file and (ii) genus‐specific FASTA files.

Mitofinder v.1.4.1 (Allio et al. 2020) was used to *de novo* assemble the trimmed reads into contigs, then scan for mitochondrial matches and annotate them, using the corresponding genus‐specific FASTA file as a reference where possible. To account for the fragmentation of mitochondrial sequences in target‐capture data, we reduced the minimum default contig size from 1000 to 500 base pairs. Mitochondrial contigs were assembled with the MEGAHIT assembler (Li et al. 2015) implemented in Mitofinder and annotated with BLAST v2.12.0, using a minimum overlap threshold of 20% and an *e*‐value cut‐off of ≤ 1e^−6^. To ensure complete annotation, we used the MitFi pipeline (Jühling et al. 2012) with the—new‐genes option to annotate any tRNAs and non‐standard animal mitochondrial genes. Additionally, the—adjust‐direction option was used to standardize the direction of the mitochondrial contigs in relation to the direction of the reference mitogenome. Following assembly and the first round of annotations in Mitofinder, only the newly generated mitochondrial genomes that contained all 13 PCGs and both rRNAs were retained for downstream analyses.

The retained mitogenomes were passed through the MitoAnnotator pipeline implemented in MitoFish v.4.09 (Iwasaki et al. 2013; Zhu et al. 2023) for a final round of annotation and circularization of the mitogenome. For mitogenomes that were fragmented across multiple contigs, all corresponding contigs were concatenated into a single file before the final round of annotations and circularization in MitoFish. Final mitochondrial assemblies were manually inspected in Geneious Prime v.2025.1.2. To resolve any tRNA duplications, only the entry with the highest tRNAscan score was retained before the mitogenome was re‐circularized. Any circularized mitogenome containing all 13 PCGs and both rRNAs, but with missing tRNAs or incomplete D‐loops, was classed as a partial mitogenome and retained for downstream phylogenetic analysis. Conversely, all circularized mitogenomes containing all the standard features of a vertebrate mitogenome were classed as complete. Subsequently, all complete mitogenomes from our study were crosschecked against all reference mitogenomes. Any newly generated mitogenome derived from a species lacking an existing reference was classified as novel. Finally, we compared the total lengths of all novel mitogenomes and the lengths of their individual components (PCGs, rRNAs, tRNAs and the D‐loop) across the well established tribe classifications in Labridae.

### 2.2 Phylogenetic Analysis

We extracted 15 mitochondrial genes, 13 PCGs and two rRNAs, from the newly generated complete and partial mitogenomes, as well as from the reference mitogenomes. For each gene, all sequences were aligned using the MAFFT v.7 aligner (Katoh and Standley 2013) with the ‐‐auto and ‐‐adjust‐direction options to determine and adjust the direction of sequences more accurately. Gene alignments were then concatenated into an alignment matrix. Phylogenetic inference was conducted with IQTree v.2 (Minh et al. 2020; Nguyen et al. 2015) using ModelFinder (Kalyaanamoorthy et al. 2017), the ‐‐rcluster 10 and the ‐m MFP + MERGE options, to identify the best‐fitting substitution models and allow merging of partitions to reduce potential model overfitting. Branch support was estimated from 1000 bootstrap replicates of the Ultrafast Bootstrap approximation (UFBoot; Hoang et al. 2018) and the SH‐like approximate likelihood ratio test (SH‐aLRT; Guindon et al. 2010). Following the IQ‐TREE manual, node values with UFBoot ≥ 95% and SH‐aLRT ≥ 80% were considered strongly supported. Nodes were considered to be moderately supported where UFBoot ranged between 70%–95%, SH‐aLRT values ranged between 70%–80%, or both values were within these respective ranges. Alternatively, low support was assigned to nodes with UFBoot or SH‐aLRT < 70%. Resulting phylogenetic reconstruction was rooted on the tribe ‘Hypsigenyinae’ based on established systematics in the family (Cowman et al. 2009; Westneat and Alfaro 2005).

## 3 Results

### 3.1 Mitogenome Assembly, Annotations and Length Comparisons

**FIGURE 2:**
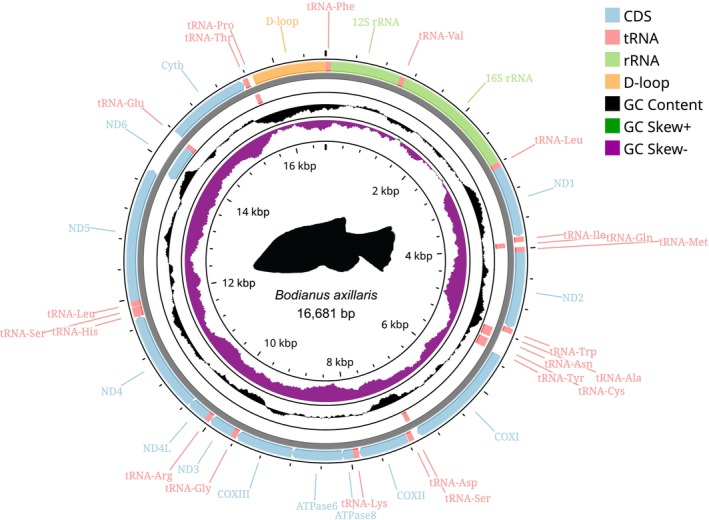
Complete novel mitochondrial genome for a labrid species— *Bodianus axillaris* (16,681 bp). The mitogenome was circularized with Proksee (Grant et al. 2023) and annotated with the complete set of 13 PCGs (blue), 2 rRNAs (green), 22 tRNAs (red) and a D‐loop/control region (orange). The L‐strand and the H‐strand of the mitochondrial DNA are represented by the inner and outer circles, respectively. The plot is also fitted with two compositional annotation tracks—GC content (in black; displayed as a deviation from the mitogenome average) and GC skew [(G–C)/(G + C)], shown as positive (green) and negative (purple) shifts around the circularized mitogenome.

Of the 388 paired‐end read libraries in the target‐capture UCE dataset (Brownstein et al. 2025) analysed in this study, we successfully extracted mitochondrial DNA from the off‐target reads of 294 libraries, accounting for more than 75% of all samples in the dataset. Across the retrieved mitochondrial data, the COX1 gene was recovered from the highest number of samples (*n* = 196; > 50%), while the Cytochrome b gene was recovered from the fewest (*n* = 149; < 40%) (see Figure S1). In total, we retrieved partial mitochondrial genes from 191 species in the family Labridae. The complete set of 15 genes was recovered from 107 different samples (> 25%; see Table S3). 32 of these possessed underlying annotation issues, such as incomplete D‐loop regions or non‐standard tRNA counts, and were classified as partial mitogenomes. The remaining 75 were classified as complete mitogenomes, representing 68 distinct labrid species. Of these, 56 mitogenomes were novel, corresponding to 54 labrid species previously lacking representative reference mitogenomes on NCBI. The remaining 19 mitogenomes matched 14 species with existing complete mitogenomes on NCBI, including three currently listed as ‘unverified’. A representative example of the complete, circularized and annotated novel mitogenomes from our study is shown in Figure 2.

**FIGURE 3:**
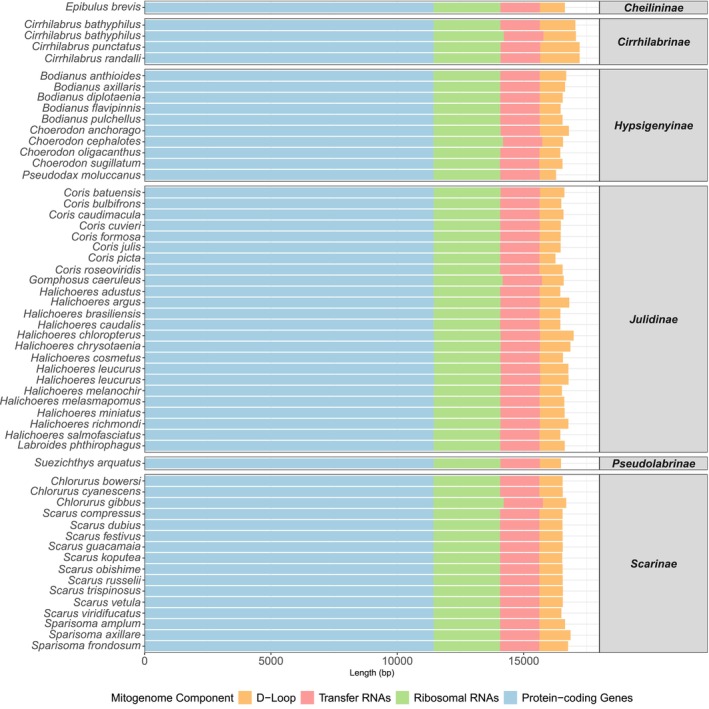
Lengths of PCGs (blue), rRNAs (green), tRNAs (red) and the D‐loop/control region (orange) across all 56 novel and complete mitogenomes, spanning 54 distinct Labrid species, retrieved in this study.

**FIGURE 4:**
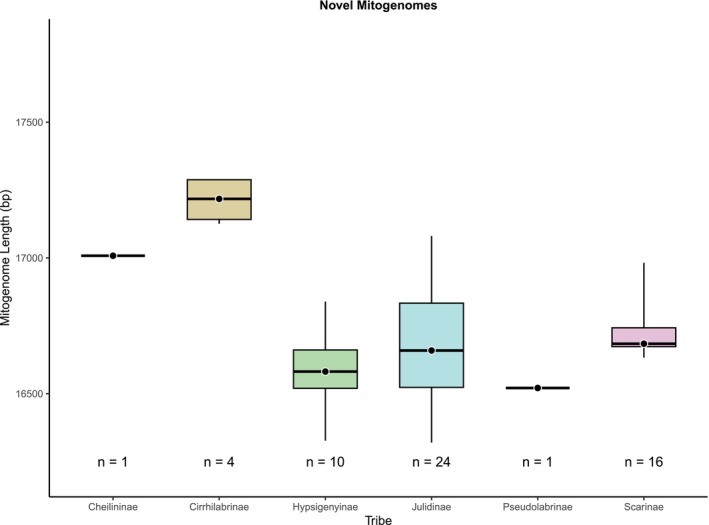
Comparison of total mitochondrial genome lengths for all 56 novel mitogenomes across their respective tribes within Labridae. Boxplots display the distribution of mitogenome lengths for each tribe, depicting the median (black dot) and all outliers (whiskers). The number of complete mitogenomes analysed per tribe is indicated below each boxplot.

The 56 novel mitogenomes span 13 genera across six tribes in the labrid tree of life: Cheilininae, Cirrhilabrinae, Hypsigenyinae, Julidinae, Pseudolabrinae and Scarinae. The lengths of all novel mitogenomes ranged from 16,320 to 17,288 bp. The composition of these mitogenomes was consistent, with the 13 PCGs spanning from 11,432 to 11,459 bp and accounting for 66.2%–70.1% of the total mitogenome length (Figure 3). The lengths of two genes—ND4L and ATPase8—were conserved across all complete mitogenomes, measuring 297 and 168 bp, respectively. The lengths of the other 11 genes, including COX1, ND2 and CytB, showed minor differences, differing only by 1–9 bp across mitogenomes (see Tables S4 and S5). The lengths of all tRNAs and both rRNAs were relatively consistent, ranging from 1548 to 1571 bp (9.1%–9.6% of the mitogenome length) and 2628 to 2783 bp (15.3%–16.6%), respectively (Figure 3). In contrast, the D‐loop exhibited substantial variation across all novel mitogenomes, ranging from 626 to 1556 bp, accounting for 3.8%–9.0% of the mitogenome (Figure 3). Species within the tribe Cirrhilabrinae had the largest D‐loop region (Figure 3) and longest mitogenomes, averaging 17,186 bp (Figure 4). Mitogenomes of species in Julidinae (*n* = 24) displayed the widest range of lengths, spanning from 16,320 (the lowest length observed among all novel mitogenomes) to 17,081 bp (Figure 4). Species within Scarinae displayed a relatively narrow range of mitogenome lengths (16,633–16,982 bp; Figure 4). Mean mitogenome lengths were comparable among Scarinae (*n* = 16; 16,737 bp), Julidinae (*n* = 24; 16,691 bp) and Hypsigenyinae (*n* = 10; 16,665 bp; Figure 4) and were greater than that of the single Pseudolabrinae representative (16,521 bp; Figure 4).

### 3.2 Phylogenetic Analysis

**FIGURE 5:**
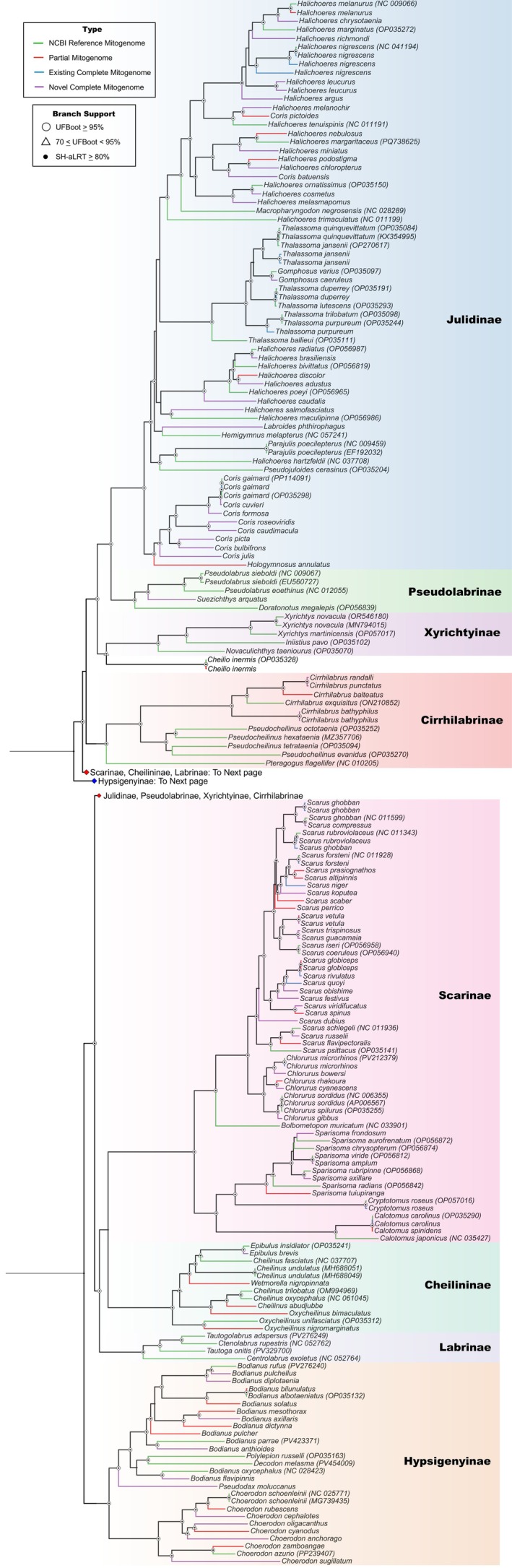
Maximum likelihood phylogenetic reconstruction for Labridae built using 107 mitochondrial sequences from our study—56 novel mitogenomes (54 species), 19 existing mitogenomes (14 species) and 32 partial mitogenomes (30 species) containing the complete set of 15 mitochondrial genes—in addition to 84 reference mitogenomes (76 species) from NCBI. Branch support was assessed with an ultrafast bootstrap approximation and the SH‐like approximate likelihood ratio test over 1000 replicates.

Final concatenated alignment matrix of 13 PCGs and two rRNAs comprised 14,708 bp and included 84 mitogenomes from the reference dataset, as well as 107 newly generated mitochondrial sequences. The latter comprised 75 complete mitogenomes—56 novel (54 unique species) and 19 existing (14 unique species)—and 32 partial mitogenomes (30 unique species). The phylogenetic reconstruction delineated eight strongly‐supported tribes (UFBoot ≥ 95, SH‐aLRT ≥ 80; Figure 5): Julidinae, Pseudolabrinae, Xyrichtyinae, Cirrhilabrinae, Scarinae, Cheilininae, Labrinae and Hypsigenyinae. Divergence event between clade 1 (comprising of Julidinae, Pseudolabrinae, Xyrichtyinae and Cirrhilabrinae) and clade 2 (comprising of Scarinae, Cheilininae and Labrinae), was also strongly supported (UFBoot/SH‐aLRT = 98.4/86). The most recent common ancestor (MRCA) to clade 1 showed moderate support (UFBoot/SH‐aLRT = 71.1/79; Figure 5). Cirrhilabrinae was an early‐diverging tribe in clade 1 (UFBoot/SH‐aLRT = 100/100; Figure 5), followed by Xyrichtyinae (UFBoot/SH‐aLRT = 100/100; Figure 5). Julidinae was recovered as the sister tribe to Pseudolabrinae with strong support (UFBoot/SH‐aLRT = 100/100; Figure 5). Clade 2 was also strongly supported (UFBoot/SH‐aLRT = 97.2/95; Figure 5) with Labrinae recovered as the early‐diverging tribe in the clade (UFBoot/SH‐aLRT = 100/100; Figure 5). However, the sister tribes in clade 2, Scarinae and Cheilininae, had low support (UFBoot/SH‐aLRT = 64.4/90; Figure 5).

## 4 Discussion

### 4.1 Mitogenome Assembly, Annotations and Length Comparisons

Of the 75 complete mitochondrial genomes extracted from the off‐target reads in the UCE targeted capture dataset, 56 were novel to the NCBI RefSeq and Nucleotide databases (see Table S3). These novel mitogenomes corresponded to 54 distinct species in Labridae, thereby increasing the total taxonomic coverage of labrid mitogenomes from less than 5% and 12% to almost 13% and 20% on the RefSeq and Nucleotide databases respectively. Each novel mitogenome from our dataset is consistent with the standard vertebrate and fish mitogenome (Pereira 2000; Satoh et al. 2016) containing 13 PCGs, two rRNAs, 22 tRNAs and a non‐coding D‐loop region.

Total lengths of all novel mitogenomes were consistent with the reference mitogenomes used in the study, ranging from 16.4 to 17.6 kbp (average: 16.8 kbp). The proportional length of all PCGs in the novel mitogenomes was also concordant with the 66.1%–69.5% observed across reference mitogenomes. Overall, the modal lengths of nine out of the 13 PCGs, in particular for ATP8 and ND4L genes, were comparable to the PCG lengths reported for 248 actinopterygian mitogenomes in Satoh et al. (2016). Only the COX3, ND2, ND3 and ND4 genes differed by 1–5 bp (see Table S4), indicating robust mitogenome assembly and annotation in the present study. Retrieved rRNA genes were also of comparable length to the reference sequences, where they comprised 15.1%–16.1% of the mitogenome. Cumulative lengths of tRNAs in novel mitogenomes had a narrower range compared to reference mitogenomes (7.8%–9.5%), suggesting slightly more consistent tRNA annotations in our dataset. Conversely, the D‐loop showed substantial differences in length across the novel and reference mitogenomes (1.9%–8.4%). This pattern is common across fish mitogenomes, as evidenced by Satoh et al. (2016) and Baraf et al. (2024), and can be attributed to the non‐coding nature of the D‐loop region that evolves under reduced selective pressure compared to PCGs.

Cirrhilabrinae had the largest mitogenomes across all labrid tribes. However, this observation is based on a small sample size of three mitogenomes and might change in the future with the increased sequencing of mitogenomes within the family. This result was, in part, inconsistent with the reference sequences, where a species in tribe Cheilininae had the longest mitogenome (17.6 kbp). However, the reference sequences of species in Hypsigenyinae, Cirrhilabrinae, Pseudolabrinae, Scarinae and Xyrichtyinae had very similar average mitogenome lengths (16.8–16.9 kbp) to those in Cheilininae (16.9 kbp), displaying the conserved nature of the total lengths of labrid mitogenomes overall.

Mitogenomes for species in Julidinae exhibited the most variation in total base pairs, agreeing with the length of reference sequences available on NCBI (16.5–17.3 kbp). However, this broad range is likely a result of taxonomic bias since Julidinae is much more speciose (*n* = 244) than any other tribes in Labridae (e.g., Labrinae, Cheilininae, Xyrichtyinae and Pseudolabrinae). Consequently, Julidinae (*n* = 25) is overrepresented in the NCBI reference dataset relative to Labrinae (*n* = 2), Pseudolabrinae (*n* = 3) and Xyrichtyinae (*n* = 4).

### 4.2 Phylogenetic Analyses

The phylogenetic tree presented in this study is, at present, the most comprehensive phylogeny based on complete mitochondrial data for the family Labridae and showed some topological discordances when compared to previously published nuclear phylogenies for the family (Brownstein et al. 2025; Hughes et al. 2023). Consistent with nuclear phylogenies, our mitochondrial phylogeny recovered Labrinae and Xyrichtyinae as the early‐diverging lineages within the Cheilininae–Scarinae and Julidinae‐Pseudolabrinae clades respectively. However, our strongly supported placement of Cirrhilabrinae within the Xyrichtyinae‐Julidinae‐Pseudolabrinae clade (UFBoot/SH‐aLRT = 100/100) was incongruent with its placement within the Labrinae‐Cheilininae‐Scarinae clade in Brownstein et al. (2025) and a majority of the maximum likelihood (ML) tree topologies in Hughes et al. (2023), revealing varying evolutionary histories for mitochondrial and nuclear genes in Labridae. Interestingly, in concordance with our phylogeny, a minority of ML tree topologies in Hughes et al. (2023) placed Cirrhilabrinae within the Xyrichtyinae‐Julidinae‐Pseudolabrinae clade, underscoring the unresolved placement of Cirrhilabrinae across different phylogenies.

Such topological discordances have been noted between trees derived from nuclear and mitochondrial markers across numerous freshwater and marine fishes (Wallis et al. 2017), including other reef fish families such as Pomacanthidae (Baraf et al. 2025). They are particularly common between phylogenies constructed using mitochondrial and nuclear markers like UCEs (Hawkins et al. 2016; Klicka et al. 2024; Salles et al. 2025; Yi et al. 2024). Incomplete lineage sorting (ILS) has often been proposed to be primary factor causing this mitonuclear discordance (Klicka et al. 2024), with studies positing that ILS is exacerbated in explosively diversifying clades, especially those with an Indo‐Pacific distribution (DeRaad et al. 2023). Since explosive diversifications have been suggested for Labridae (Brownstein et al. 2025), with the Indo‐Australian archipelago (IAA) being a biodiversity hotspot for several labrid lineages (Barber and Bellwood 2005; Puckridge et al. 2015; Read et al. 2006), we hypothesize that ILS is likely one of the driving factors behind this mitonuclear discordance in this family. Other factors like interspecific hybridization, which can lead to mitochondrial introgression (Wallis et al. 2017), have also been suggested as determinants of mitonuclear discordance (Linnen and Farrell 2007). Interspecific hybrids are prevalent in coral reef ecosystems (Hobbs et al. 2022), including in prominent reef fish families like Chaetodontidae and Pomacanthidae (Yaakub et al. 2006), and they have also been documented in Labridae (Ayling 1980; Yaakub et al. 2007, 2006), indicating that hybridization and mitochondrial introgression might be contributing factors to the mitonuclear discordance in this family. However, due to the maternally‐inherited (Hutchison III et al. 1974) and generally non‐recombining nature (Galtier et al. 2009) of mitochondrial DNA, the mitogenome behaves as a single locus (Main et al. 2024; Stelbrink et al. 2024). Since the determinants of mitonuclear discordance can be masked when studying only a single genomic marker, we recommend an integrative approach that combines the mitochondrial data with multi‐locus genomic datasets such as UCEs to enhance our understanding of the effects of ILS, interspecific hybridization and mitochondrial introgression on the discordance among Labrid phylogenies, further disentangling the complex evolutionary history within the Labridae tree of life.

## 5 Conclusion

This study substantially expands the mitochondrial genomic resources available for Labridae by generating novel mitogenomes for 54 unique species, increasing the family's mitogenomic representation from 32 and 81 labrid species (5% and 12% of the total family) to 86 and 135 labrid species (13% and 20% of the total family) on NCBI's RefSeq and Nucleotide databases, respectively. Additionally, we also generated partial mitogenomic data for a further 191 unique species in the family. Length and composition of labrid mitogenomes were highly consistent across datasets and with published actinopterygian mitochondrial data, with the D‐loop region exhibiting the most variation in total base pairs. Tribe‐wise comparisons revealed potential taxonomic differences in mitogenome length (e.g., Julidinae).

The eight major labrid tribes were strongly supported by maximum likelihood analysis based on mitochondrial data and branching arrangements were mostly congruent with previous topologies inferred from nuclear markers. However, the position of Cirrhilabrinae differed, highlighting mitonuclear discordances for the family that might reflect underlying processes such as incomplete lineage sorting, mitochondrial introgression or interspecific hybridization. Finally, this study shows that mitochondrial data can resolve broad phylogenetic relationships within Labridae and help diagnose sources of phylogenetic discordance. Future research should, therefore, combine mitochondrial and TCS nuclear datasets to advance understanding of the complex evolutionary history of Labridae. The high‐quality mitogenomic resources reported in the present study provide a valuable foundation for future research on the phylogenetics, molecular evolution and eDNA monitoring studies of this speciose reef fish family.

## Author Contributions

**Aditya V. Swami:** conceptualization (lead), data curation (lead), formal analysis (lead), investigation (lead), methodology (lead), visualization (lead), writing – original draft (lead). **Lauriane M. Baraf:** supervision (supporting), writing – review and editing (supporting). **Peter F. Cowman:** conceptualization (supporting), funding acquisition (lead), resources (lead), supervision (lead), writing – review and editing (lead).

## Conflicts of Interest

The authors declare no conflicts of interest.

## Supporting information


**Data S1:** ece373270‐sup‐0001‐Supinfo.docx.

## Data Availability

Bioinformatic scripts are available on GitHub (https://github.com/aditya‐jv9/Labrid‐Mitogenomes). All gene alignments and treefiles generated during the course of this study are made available publicly on Figshare (https://figshare.com/articles/dataset/Treefiles_and_Alignments_for_Mining_for_mitochondria_68_mitogenomes_for_wrasses_and_parrotfishes_F_Labridae_from_off‐target_UCE_data_/29832998). All novel mitogenomes assembled and annotated in the present study are made available on the NCBI GenBank Database (BioProject PRJNA1335850).
