# Supplementary material for: Mining for Mitochondria: 68 Mitogenomes for Wrasses and Parrotfishes (F: Labridae) From Off‐Target UCE Data
Source: Ecol Evol. 2026 Mar 18;16(3):e73270. doi: 10.1002/ece3.73270 (PMC13093266; doi:10.1002/ece3.73270)
Supplement: Supplementary file 1 — Data S1: ece373270‐sup‐0001‐Supinfo.docx. [file ECE3-16-e73270-s001.docx]

Mining for mitochondria: 68 mitogenomes for wrasses and parrotfishes (F: Labridae) from off-target UCE data – Supplementary Data

*Supplementary Table 1 –* Accession numbers, species ID and the authors for all reference mitogenomes downloaded from NCBI during this study.

| **Accession** | **Species** | **Author** |
| --- | --- | --- |
| OP035132 | *Bodianus albotaeniatus* | Bemis et al., 2023 |
| KT591189 | *Bodianus oxycephalus* | Wang et al., 2015 |
| NC 028423 | *Bodianus oxycephalus* | Wang et al., 2023 |
| PV423371 | *Bodianus parrae* | Bemis et al., 2025 |
| PV276240 | *Bodianus rufus* | Bemis et al., 2025 |
| KY235362 | *Bolbometopon muricatum* | Jang-Liaw et al., 2017 |
| NC 033901 | *Bolbometopon muricatum* | Jang-Liaw et al., 2023 |
| OP035290 | *Calotomus carolinus* | Bemis et al., 2023 |
| NC 035427 | *Calotomus japonicus* | Mabuchi et al., 2023 |
| MT410926 | *Centrolabrus exoletus* | Margaryan et al., 2020 |
| NC 052764 | *Centrolabrus exoletus* | Margaryan et al., 2023 |
| KY115687 | *Cheilinus fasciatus* | Zhang et al., 2018 |
| NC 037707 | *Cheilinus fasciatus* | Zhang et al., 2023 |
| MN399860 | *Cheilinus oxycephalus* | Guo et al., 2022 |
| NC 061045 | *Cheilinus oxycephalus* | Guo et al., 2023 |
| NC 065150 | *Cheilinus trilobatus* | Wang et al., 2023 |
| OM994969 | *Cheilinus trilobatus* | Wang et al., 2022 |
| GU296101 | *Cheilinus undulatus* | Qi et al., 2013 |
| KM461717 | *Cheilinus undulatus* | Han et al., 2014 |
| MH675879 | *Cheilinus undulatus* | Matthew et al., 2019 |
| MH675880 | *Cheilinus undulatus* | Matthew et al., 2019 |
| MH688049 | *Cheilinus undulatus* | Matthew et al., 2019 |
| MH688050 | *Cheilinus undulatus* | Matthew et al., 2019 |
| MH688051 | *Cheilinus undulatus* | Matthew et al., 2019 |
| MH688052 | *Cheilinus undulatus* | Matthew et al., 2019 |
| NC 013842 | *Cheilinus undulatus* | Yin et al., 2023 |
| OP035328 | *Cheilio inermis* | Bemis et al., 2023 |
| PV212379 | *Chlorurus microrhinos* | Gao et al., 2025 |
| AP006567 | *Chlorurus sordidus* | Mabuchi et al., 2016 |
| NC 006355 | *Chlorurus sordidus* | Mabuchi et al., 2023 |
| OP035255 | *Chlorurus spilurus* | Bemis et al., 2023 |
| NC 086888 | *Choerodon azurio* | Zhu et al., 2024 |
| PP239407 | *Choerodon azurio* | Zhu et al., 2024 |
| KM487697 | *Choerodon schoenleinii* | Wen et al., 2014 |
| MG739435 | *Choerodon schoenleinii* | Du et al., 2019 |
| NC 025771 | *Choerodon schoenleinii* | Wen et al., 2023 |
| NC 064113 | *Cirrhilabrus exquisitus* | Shan et al., 2023 |
| ON210852 | *Cirrhilabrus exquisitus* | Shan et al., 2022 |

| OP035298 | *Coris gaimard* | Bemis et al., 2023 |
| --- | --- | --- |
| PP114091 | *Coris gaimard* | Wang et al., 2024 |
| OP057016 | *Cryptotomus roseus* | Bemis et al., 2023 |
| MT410914 | *Ctenolabrus rupestris* | Margaryan et al., 2020 |
| NC 052762 | *Ctenolabrus rupestris* | Margaryan et al., 2023 |
| PV454009 | *Decodon melasma* | Bemis et al., 2025 |
| OP056839 | *Doratonotus megalepis* | Bemis et al., 2023 |
| OP035241 | *Epibulus insidiator* | Bemis et al., 2023 |
| OP035097 | *Gomphosus varius* | Bemis et al., 2023 |
| OP056819 | *Halichoeres bivittatus* | Bemis et al., 2023 |
| KY115688 | *Halichoeres hartzfeldii* | Zhang et al., 2018 |
| NC 037708 | *Halichoeres hartzfeldii* | Zhang et al., 2023 |
| OP056986 | *Halichoeres maculipinna* | Bemis et al., 2023 |
| PQ738625 | *Halichoeres margaritaceus* | Huang et al., 2025 |
| OP035272 | *Halichoeres marginatus* | Bemis et al., 2023 |
| AP006018 | *Halichoeres melanurus* | Mabuchi et al., 2016 |
| NC 009066 | *Halichoeres melanurus* | Mabuchi et al., 2023 |
| MH678616 | *Halichoeres nigrescens* | Shi et al., 2019 |
| NC 041194 | *Halichoeres nigrescens* | Shi et al., 2023 |
| OP035150 | *Halichoeres ornatissimus* | Bemis et al., 2023 |
| OP056965 | *Halichoeres poeyi* | Bemis et al., 2023 |
| OP056987 | *Halichoeres radiatus* | Bemis et al., 2023 |
| EU082205 | *Halichoeres tenuispinis* | Oh et al., 2016 |
| NC 011191 | *Halichoeres tenuispinis* | Oh et al., 2023 |
| EU087704 | *Halichoeres trimaculatus* | Oh et al., 2016 |
| NC 011199 | *Halichoeres trimaculatus* | Oh et al., 2023 |
| MN614148 | *Hemigymnus melapterus* | Yi et al., 2021 |
| NC 057241 | *Hemigymnus melapterus* | Yi et al., 2023 |
| OP035102 | *Iniistius pavo* | Bemis et al., 2023 |
| KP013102 | *Macropharyngodon negrosensis* | Renshaw et al., 2015 |
| NC 028289 | *Macropharyngodon negrosensis* | Renshaw et al., 2023 |
| OP035070 | *Novaculichthys taeniourus* | Bemis et al., 2023 |
| OP035312 | *Oxycheilinus unifasciatus* | Bemis et al., 2023 |
| EF192032 | *Parajulis poecilepterus* | Oh et al., 2016 |
| NC 009459 | *Parajulis poecilepterus* | Oh et al., 2023 |
| OP035163 | *Polylepion russelli* | Bemis et al., 2023 |
| OP035270 | *Pseudocheilinus evanidus* | Bemis et al., 2023 |
| MZ357706 | *Pseudocheilinus hexataenia* | Eom et al., 2021 |
| OP035252 | *Pseudocheilinus octotaenia* | Bemis et al., 2023 |
| OP035094 | *Pseudocheilinus tetrataenia* | Bemis et al., 2023 |
| OP035204 | *Pseudojuloides cerasinus* | Bemis et al., 2023 |
| EU560728 | *Pseudolabrus eoethinus* | Oh et al., 2016 |
| NC 012055 | *Pseudolabrus eoethinus* | Oh et al., 2023 |
| AP006019 | *Pseudolabrus sieboldi* | Mabuchi et al., 2016 |
| EU560727 | *Pseudolabrus sieboldi* | Oh et al., 2016 |

| NC 009067 | *Pseudolabrus sieboldi* | Mabuchi et al., 2023 |
| --- | --- | --- |
| EF409976 | *Pteragogus flagellifer* | Jung et al., 2016 |
| NC 010205 | *Pteragogus flagellifer* | Jung et al., 2023 |
| OP056940 | *Scarus coeruleus* | Bemis et al., 2023 |
| FJ619271 | *Scarus forsteni* | Tang et al., 2016 |
| NC 011928 | *Scarus forsteni* | Tang et al., 2023 |
| FJ449707 | *Scarus ghobban* | Cheng et al., 2016 |
| NC 011599 | *Scarus ghobban* | Cheng et al., 2023 |
| OP056958 | *Scarus iseri* | Bemis et al., 2023 |
| OP035141 | *Scarus psittacus* | Bemis et al., 2023 |
| FJ227899 | *Scarus rubroviolaceus* | Cheng et al., 2016 |
| NC 011343 | *Scarus rubroviolaceus* | Cheng et al., 2023 |
| FJ595020 | *Scarus schlegeli* | Tang et al., 2016 |
| NC 011936 | *Scarus schlegeli* | Tang et al., 2023 |
| OP056872 | *Sparisoma aurofrenatum* | Bemis et al., 2023 |
| OP056874 | *Sparisoma chrysopterum* | Bemis et al., 2023 |
| OP056842 | *Sparisoma radians* | Bemis et al., 2023 |
| OP056868 | *Sparisoma rubripinne* | Bemis et al., 2023 |
| OP056812 | *Sparisoma viride* | Bemis et al., 2023 |
| PV329700 | *Tautoga onitis* | Bemis et al., 2025 |
| PV276249 | *Tautogolabrus adspersus* | Bemis et al., 2025 |
| OP035111 | *Thalassoma ballieui* | Bemis et al., 2023 |
| OP035191 | *Thalassoma duperrey* | Bemis et al., 2023 |
| NC 071873 | *Thalassoma jansenii* | Zhao et al., 2023 |
| OP270617 | *Thalassoma jansenii* | Zhao et al., 2023 |
| MN170516 | *Thalassoma lunare* | Yang et al., 2020 |
| NC 048980 | *Thalassoma lunare* | Yang et al., 2023 |
| OP035293 | *Thalassoma lutescens* | Bemis et al., 2023 |
| OP035244 | *Thalassoma purpureum* | Bemis et al., 2023 |
| KX354995 | *Thalassoma quinquevittatum* | Zhang et al., 2016 |
| OP035084 | *Thalassoma quinquevittatum* | Bemis et al., 2023 |
| OP035098 | *Thalassoma trilobatum* | Bemis et al., 2023 |
| OP057017 | *Xyrichtys martinicensis* | Bemis et al., 2023 |
| MN794015 | *Xyrichtys novacula* | Barcelo-Serra et al., 2020 |
| NC 083028 | *Xyrichtys novacula* | Bemis et al., 2023 |
| OR546180 | *Xyrichtys novacula* | Bemis et al., 2023 |

*Supplementary Table 2 –* Accession numbers, species ID and the authors for all duplicate reference mitogenomes not included in the phylogenetic reconstruction*.*

| **Accession** | **Species** | **Author** |
| --- | --- | --- |
| KT591189 | *Bodianus oxycephalus* | Wang et al., 2015 |
| KY235362 | *Bolbometopon muricatum* | Jang-Liaw et al., 2017 |
| MT410926 | *Centrolabrus exoletus* | Margaryan et al., 2020 |
| KY115687 | *Cheilinus fasciatus* | Zhang et al., 2018 |
| MN399860 | *Cheilinus oxycephalus* | Guo et al., 2022 |
| NC 065150 | *Cheilinus trilobatus* | Wang et al., 2023 |
| GU296101 | *Cheilinus undulatus* | Qi et al., 2013 |
| KM461717 | *Cheilinus undulatus* | Han et al., 2014 |
| MH675879 | *Cheilinus undulatus* | Matthew et al., 2019 |
| MH675880 | *Cheilinus undulatus* | Matthew et al., 2019 |
| MH688050 | *Cheilinus undulatus* | Matthew et al., 2019 |
| MH688052 | *Cheilinus undulatus* | Matthew et al., 2019 |
| NC 013842 | *Cheilinus undulatus* | Yin et al., 2023 |
| NC 086888 | *Choerodon azurio* | Zhu et al., 2024 |
| KM487697 | *Choerodon schoenleinii* | Wen et al., 2014 |
| NC 064113 | *Cirrhilabrus exquisitus* | Shan et al., 2023 |
| MT410914 | *Ctenolabrus rupestris* | Margaryan et al., 2020 |
| KY115688 | *Halichoeres hartzfeldii* | Zhang et al., 2018 |
| AP006018 | *Halichoeres melanurus* | Mabuchi et al., 2016 |
| MH678616 | *Halichoeres nigrescens* | Shi et al., 2019 |
| EU082205 | *Halichoeres tenuispinis* | Oh et al., 2016 |
| EU087704 | *Halichoeres trimaculatus* | Oh et al., 2016 |
| MN614148 | *Hemigymnus melapterus* | Yi et al., 2021 |
| KP013102 | *Macropharyngodon negrosensis* | Renshaw et al., 2015 |
| EU560728 | *Pseudolabrus eoethinus* | Oh et al., 2016 |
| AP006019 | *Pseudolabrus sieboldi* | Mabuchi et al., 2016 |
| EF409976 | *Pteragogus flagellifer* | Jung et al., 2016 |
| FJ619271 | *Scarus forsteni* | Tang et al., 2016 |
| FJ449707 | *Scarus ghobban* | Cheng et al., 2016 |
| FJ227899 | *Scarus rubroviolaceus* | Cheng et al., 2016 |
| FJ595020 | *Scarus schlegeli* | Tang et al., 2016 |
| NC 071873 | *Thalassoma jansenii* | Zhao et al., 2023 |
| MN170516 | *Thalassoma lunare* | Yang et al., 2020 |
| NC 048980 | *Thalassoma lunare* | Yang et al., 2023 |
| NC 083028 | *Xyrichtys novacula* | Bemis et al., 2023 |

*Supplementary Table 3 –* Species, classification and clade designation for the 107 assemblies from our study with the complete mitochondrial set of 13 PCGs and two rRNAs used for the mitochondrial phylogenetic reconstruction

| **Species** | **Classification** | **Clade** |
| --- | --- | --- |
| *Bodianus anthioides* | Novel Complete Mitogenome | Hypsigenyinae |
| *Bodianus axillaris* | Novel Complete Mitogenome | Hypsigenyinae |
| *Bodianus bilunulatus* | Partial Mitogenome (13 PCGs and 2 rRNAs) | Hypsigenyinae |
| *Bodianus dictynna* | Partial Mitogenome (13 PCGs and 2 rRNAs) | Hypsigenyinae |
| *Bodianus diplotaenia* | Novel Complete Mitogenome | Hypsigenyinae |
| *Bodianus flavipinnis* | Novel Complete Mitogenome | Hypsigenyinae |
| *Bodianus mesothorax* | Partial Mitogenome (13 PCGs and 2 rRNAs) | Hypsigenyinae |
| *Bodianus pulchellus* | Novel Complete Mitogenome | Hypsigenyinae |
| *Bodianus solatus* | Partial Mitogenome (13 PCGs and 2 rRNAs) | Hypsigenyinae |
| *Calotomus carolinus* | Existing Complete Mitogenome | Scarinae |
| *Calotomus spinidens* | Novel Complete Mitogenome | Scarinae |
| *Cheilinus abudjubbe* | Partial Mitogenome (13 PCGs and 2 rRNAs) | Cheilininae |
| *Cheilio inermis* | Partial Mitogenome (13 PCGs and 2 rRNAs) |  |
| *Chlorurus bowersi* | Novel Complete Mitogenome | Scarinae |
| *Chlorurus cyanescens* | Novel Complete Mitogenome | Scarinae |
| *Chlorurus gibbus* | Novel Complete Mitogenome | Scarinae |
| *Chlorurus microrhinos* | Existing Complete Mitogenome | Scarinae |
| *Chlorurus rhakoura* | Partial Mitogenome (13 PCGs and 2 rRNAs) | Scarinae |
| *Choerodon anchorago* | Novel Complete Mitogenome | Hypsigenyinae |
| *Choerodon cephalotes* | Novel Complete Mitogenome | Hypsigenyinae |
| *Choerodon cyanodus* | Partial Mitogenome (13 PCGs and 2 rRNAs) | Hypsigenyinae |
| *Choerodon oligacanthus* | Novel Complete Mitogenome | Hypsigenyinae |
| *Choerodon rubescens* | Partial Mitogenome (13 PCGs and 2 rRNAs) | Hypsigenyinae |
| *Choerodon sugillatum* | Novel Complete Mitogenome | Hypsigenyinae |
| *Choerodon zamboangae* | Partial Mitogenome (13 PCGs and 2 rRNAs) | Hypsigenyinae |
| *Cirrhilabrus balteatus* | Partial Mitogenome (13 PCGs and 2 rRNAs) | Cirrhilabrinae |
| *Cirrhilabrus bathyphilus* | Novel Complete Mitogenome | Cirrhilabrinae |
| *Cirrhilabrus bathyphilus* | Novel Complete Mitogenome | Cirrhilabrinae |
| *Cirrhilabrus punctatus* | Novel Complete Mitogenome | Cirrhilabrinae |
| *Cirrhilabrus randalli* | Novel Complete Mitogenome | Cirrhilabrinae |
| *Coris batuensis* | Novel Complete Mitogenome | Julidinae |
| *Coris bulbifrons* | Novel Complete Mitogenome | Julidinae |
| *Coris caudimacula* | Novel Complete Mitogenome | Julidinae |
| *Coris cuvieri* | Novel Complete Mitogenome | Julidinae |
| *Coris formosa* | Novel Complete Mitogenome | Julidinae |
| *Coris gaimard* | Existing Complete Mitogenome | Julidinae |
| *Coris julis* | Novel Complete Mitogenome | Julidinae |
| *Coris picta* | Novel Complete Mitogenome | Julidinae |
| *Coris pictoides* | Partial Mitogenome (13 PCGs and 2 rRNAs) | Julidinae |
| *Coris roseoviridis* | Novel Complete Mitogenome | Julidinae |

| *Cryptotomus roseus* | Existing Complete Mitogenome | Scarinae |
| --- | --- | --- |
| *Epibulus brevis* | Novel Complete Mitogenome | Cheilininae |
| *Gomphosus caeruleus* | Novel Complete Mitogenome | Julidinae |
| *Halichoeres adustus* | Novel Complete Mitogenome | Julidinae |
| *Halichoeres argus* | Novel Complete Mitogenome | Julidinae |
| *Halichoeres brasiliensis* | Novel Complete Mitogenome | Julidinae |
| *Halichoeres caudalis* | Novel Complete Mitogenome | Julidinae |
| *Halichoeres chloropterus* | Novel Complete Mitogenome | Julidinae |
| *Halichoeres chrysotaenia* | Novel Complete Mitogenome | Julidinae |
| *Halichoeres cosmetus* | Novel Complete Mitogenome | Julidinae |
| *Halichoeres discolor* | Partial Mitogenome (13 PCGs and 2 rRNAs) | Julidinae |
| *Halichoeres nigrescens* | Existing Complete Mitogenome | Julidinae |
| *Halichoeres nigrescens* | Existing Complete Mitogenome | Julidinae |
| *Halichoeres leucurus* | Novel Complete Mitogenome | Julidinae |
| *Halichoeres melanochir* | Novel Complete Mitogenome | Julidinae |
| *Halichoeres melanurus* | Partial Mitogenome (13 PCGs and 2 rRNAs) | Julidinae |
| *Halichoeres melasmapomus* | Novel Complete Mitogenome | Julidinae |
| *Halichoeres miniatus* | Novel Complete Mitogenome | Julidinae |
| *Halichoeres nebulosus* | Partial Mitogenome (13 PCGs and 2 rRNAs) | Julidinae |
| *Halichoeres nigrescens* | Existing Complete Mitogenome | Julidinae |
| *Halichoeres podostigma* | Partial Mitogenome (13 PCGs and 2 rRNAs) | Julidinae |
| *Halichoeres leucurus* | Novel Complete Mitogenome | Julidinae |
| *Halichoeres richmondi* | Novel Complete Mitogenome | Julidinae |
| *Halichoeres salmofasciatus* | Novel Complete Mitogenome | Julidinae |
| *Hologymnosus annulatus* | Partial Mitogenome (13 PCGs and 2 rRNAs) | Julidinae |
| *Labroides phthirophagus* | Novel Complete Mitogenome | Julidinae |
| *Oxycheilinus bimaculatus* | Partial Mitogenome (13 PCGs and 2 rRNAs) | Cheilininae |
| *Oxycheilinus nigromarginatus* | Partial Mitogenome (13 PCGs and 2 rRNAs) | Cheilininae |
| *Pseudodax moluccanus* | Novel Complete Mitogenome | Hypsigenyinae |
| *Scarus altipinnis* | Partial Mitogenome (13 PCGs and 2 rRNAs) | Scarinae |
| *Scarus compressus* | Novel Complete Mitogenome | Scarinae |
| *Scarus dubius* | Novel Complete Mitogenome | Scarinae |
| *Scarus festivus* | Novel Complete Mitogenome | Scarinae |
| *Scarus flavipectoralis* | Partial Mitogenome (13 PCGs and 2 rRNAs) | Scarinae |
| *Scarus forsteni* | Existing Complete Mitogenome | Scarinae |
| *Scarus ghobban* | Existing Complete Mitogenome | Scarinae |
| *Scarus ghobban* | Existing Complete Mitogenome | Scarinae |
| *Scarus ghobban* | Existing Complete Mitogenome | Scarinae |
| *Scarus globiceps* | Partial Mitogenome (13 PCGs and 2 rRNAs) | Scarinae |
| *Scarus globiceps* | Partial Mitogenome (13 PCGs and 2 rRNAs) | Scarinae |
| *Scarus guacamaia* | Novel Complete Mitogenome | Scarinae |
| *Scarus koputea* | Novel Complete Mitogenome | Scarinae |
| *Scarus niger* | Existing Complete Mitogenome | Scarinae |
| *Scarus obishime* | Novel Complete Mitogenome | Scarinae |

| *Scarus perrico* | Partial Mitogenome (13 PCGs and 2 rRNAs) | Scarinae |
| --- | --- | --- |
| *Scarus prasiognathos* | Partial Mitogenome (13 PCGs and 2 rRNAs) | Scarinae |
| *Scarus quoyi* | Existing Complete Mitogenome | Scarinae |
| *Scarus rivulatus* | Existing Complete Mitogenome | Scarinae |
| *Scarus rubroviolaceus* | Existing Complete Mitogenome | Scarinae |
| *Scarus russelii* | Novel Complete Mitogenome | Scarinae |
| *Scarus scaber* | Partial Mitogenome (13 PCGs and 2 rRNAs) | Scarinae |
| *Scarus spinus* | Partial Mitogenome (13 PCGs and 2 rRNAs) | Scarinae |
| *Scarus trispinosus* | Novel Complete Mitogenome | Scarinae |
| *Scarus vetula* | Novel Complete Mitogenome | Scarinae |
| *Scarus vetula* | Partial Mitogenome (13 PCGs and 2 rRNAs) | Scarinae |
| *Scarus viridifucatus* | Novel Complete Mitogenome | Scarinae |
| *Bodianus pulcher* | Partial Mitogenome (13 PCGs and 2 rRNAs) | Hypsigenyinae |
| *Sparisoma amplum* | Novel Complete Mitogenome | Scarinae |
| *Sparisoma axillare* | Novel Complete Mitogenome | Scarinae |
| *Sparisoma frondosum* | Novel Complete Mitogenome | Scarinae |
| *Sparisoma tuiupiranga* | Partial Mitogenome (13 PCGs and 2 rRNAs) | Scarinae |
| *Suezichthys arquatus* | Novel Complete Mitogenome | Pseudolabrinae |
| *Thalassoma duperrey* | Existing Complete Mitogenome | Julidinae |
| *Thalassoma jansenii* | Existing Complete Mitogenome | Julidinae |
| *Thalassoma jansenii* | Existing Complete Mitogenome | Julidinae |
| *Thalassoma purpureum* | Existing Complete Mitogenome | Julidinae |
| *Wetmorella nigropinnata* | Partial Mitogenome (13 PCGs and 2 rRNAs) | Cheilininae |

*Supplementary Table 4 –* Lengths of individual PCGs across all 56 novel complete mitogenomes generated in this study

| **Species** | **COI** | **COII** | **COIII** | **ND1** | **ND2** | **ND3** | **ND4** | **ND4L** | **ND5** | **ND6** | **ATP6** | **ATP8** | **CYTB** |
| --- | --- | --- | --- | --- | --- | --- | --- | --- | --- | --- | --- | --- | --- |
| *Cirrhilabrus punctatus* | 1554 | 691 | 786 | 975 | 1047 | 354 | 1381 | 297 | 1839 | 522 | 684 | 168 | 1141 |
| *Cirrhilabrus randalli* | 1554 | 691 | 786 | 975 | 1047 | 354 | 1381 | 297 | 1839 | 522 | 684 | 168 | 1141 |
| *Cirrhilabrus bathyphilus* | 1554 | 691 | 786 | 975 | 1047 | 354 | 1381 | 297 | 1839 | 522 | 684 | 168 | 1141 |
| *Halichoeres chloropterus* | 1560 | 691 | 786 | 975 | 1047 | 354 | 1380 | 297 | 1839 | 522 | 684 | 168 | 1141 |
| *Cirrhilabrus bathyphilus* | 1554 | 691 | 786 | 975 | 1047 | 354 | 1381 | 297 | 1839 | 522 | 684 | 168 | 1141 |
| *Sparisoma axillare* | 1551 | 691 | 786 | 975 | 1047 | 354 | 1381 | 297 | 1848 | 522 | 684 | 168 | 1141 |
| *Halichoeres chrysotaenia* | 1560 | 691 | 786 | 975 | 1047 | 354 | 1380 | 297 | 1839 | 531 | 684 | 168 | 1141 |
| *Halichoeres argus* | 1560 | 691 | 786 | 975 | 1047 | 354 | 1380 | 297 | 1839 | 531 | 684 | 168 | 1141 |
| *Choerodon anchorago* | 1551 | 691 | 786 | 975 | 1050 | 351 | 1381 | 297 | 1839 | 522 | 684 | 168 | 1141 |
| *Sparisoma frondosum* | 1551 | 691 | 786 | 975 | 1047 | 354 | 1381 | 297 | 1848 | 522 | 678 | 168 | 1141 |
| *Halichoeres richmondi* | 1560 | 691 | 786 | 975 | 1047 | 354 | 1380 | 297 | 1839 | 531 | 684 | 168 | 1141 |
| *Halichoeres leucurus* | 1560 | 691 | 786 | 975 | 1047 | 354 | 1380 | 297 | 1839 | 531 | 684 | 168 | 1141 |
| *Halichoeres leucurus* | 1560 | 697 | 786 | 975 | 1047 | 354 | 1380 | 297 | 1839 | 531 | 684 | 168 | 1141 |
| *Bodianus anthioides* | 1551 | 691 | 786 | 975 | 1047 | 351 | 1381 | 297 | 1839 | 522 | 684 | 168 | 1141 |
| *Sparisoma amplum* | 1551 | 691 | 786 | 975 | 1047 | 354 | 1381 | 297 | 1848 | 522 | 684 | 168 | 1141 |
| *Bodianus axillaris* | 1551 | 691 | 786 | 975 | 1047 | 351 | 1381 | 297 | 1839 | 522 | 684 | 168 | 1141 |
| *Labroides phthirophagus* | 1551 | 691 | 786 | 975 | 1047 | 354 | 1386 | 297 | 1839 | 522 | 684 | 168 | 1141 |
| *Epibulus brevis* | 1551 | 691 | 786 | 975 | 1047 | 352 | 1386 | 297 | 1839 | 522 | 684 | 168 | 1141 |
| *Halichoeres miniatus* | 1560 | 691 | 786 | 975 | 1047 | 354 | 1380 | 297 | 1833 | 522 | 684 | 168 | 1141 |
| *Halichoeres melasmapomus* | 1560 | 691 | 786 | 975 | 1047 | 354 | 1380 | 297 | 1839 | 522 | 684 | 168 | 1141 |
| *Coris batuensis* | 1560 | 691 | 786 | 975 | 1047 | 354 | 1380 | 297 | 1839 | 522 | 684 | 168 | 1143 |
| *Coris caudimacula* | 1551 | 691 | 786 | 975 | 1047 | 354 | 1381 | 297 | 1839 | 522 | 684 | 168 | 1141 |
| *Scarus trispinosus* | 1551 | 691 | 786 | 972 | 1047 | 354 | 1380 | 297 | 1839 | 522 | 684 | 168 | 1141 |
| *Scarus vetula* | 1551 | 691 | 786 | 972 | 1047 | 354 | 1380 | 297 | 1839 | 522 | 684 | 168 | 1141 |
| *Scarus guacamaia* | 1551 | 691 | 786 | 972 | 1047 | 354 | 1380 | 297 | 1839 | 522 | 684 | 168 | 1141 |

| *Choerodon sugillatum* | 1551 | 691 | 786 | 975 | 1047 | 351 | 1381 | 297 | 1839 | 522 | 684 | 168 | 1141 |
| --- | --- | --- | --- | --- | --- | --- | --- | --- | --- | --- | --- | --- | --- |
| *Halichoeres cosmetus* | 1560 | 691 | 786 | 975 | 1047 | 354 | 1380 | 297 | 1839 | 522 | 684 | 168 | 1141 |
| *Chlorurus cyanescens* | 1551 | 691 | 786 | 972 | 1047 | 354 | 1380 | 297 | 1839 | 522 | 684 | 168 | 1141 |
| *Scarus russelii* | 1551 | 691 | 786 | 972 | 1047 | 354 | 1380 | 297 | 1839 | 522 | 684 | 168 | 1141 |
| *Scarus festivus* | 1551 | 691 | 786 | 972 | 1047 | 354 | 1380 | 297 | 1839 | 522 | 684 | 168 | 1141 |
| *Scarus obishime* | 1551 | 691 | 786 | 972 | 1047 | 354 | 1380 | 297 | 1839 | 522 | 684 | 168 | 1141 |
| *Chlorurus gibbus* | 1551 | 691 | 786 | 972 | 1047 | 354 | 1380 | 297 | 1839 | 522 | 684 | 168 | 1141 |
| *Chlorurus bowersi* | 1551 | 691 | 786 | 972 | 1047 | 354 | 1380 | 297 | 1839 | 522 | 684 | 168 | 1141 |
| *Coris roseoviridis* | 1551 | 691 | 786 | 975 | 1045 | 354 | 1381 | 297 | 1839 | 522 | 684 | 168 | 1141 |
| *Scarus compressus* | 1551 | 691 | 786 | 972 | 1047 | 354 | 1380 | 297 | 1839 | 522 | 684 | 168 | 1141 |
| *Scarus koputea* | 1551 | 691 | 786 | 972 | 1047 | 354 | 1380 | 297 | 1839 | 522 | 684 | 168 | 1141 |
| *Scarus dubius* | 1551 | 691 | 786 | 972 | 1047 | 354 | 1380 | 297 | 1839 | 522 | 684 | 168 | 1141 |
| *Bodianus diplotaenia* | 1551 | 691 | 786 | 975 | 1047 | 351 | 1381 | 297 | 1839 | 522 | 684 | 168 | 1141 |
| *Bodianus pulchellus* | 1551 | 691 | 785 | 975 | 1047 | 351 | 1381 | 297 | 1839 | 522 | 684 | 168 | 1141 |
| *Halichoeres melanochir* | 1560 | 691 | 786 | 975 | 1047 | 354 | 1380 | 297 | 1836 | 522 | 684 | 168 | 1141 |
| *Scarus viridifucatus* | 1551 | 691 | 786 | 972 | 1047 | 354 | 1380 | 297 | 1839 | 522 | 684 | 168 | 1141 |
| *Gomphosus caeruleus* | 1551 | 691 | 786 | 975 | 1047 | 354 | 1380 | 297 | 1839 | 522 | 684 | 168 | 1141 |
| *Coris bulbifrons* | 1551 | 691 | 786 | 975 | 1047 | 354 | 1381 | 297 | 1842 | 522 | 684 | 168 | 1141 |
| *Suezichthys arquatus* | 1551 | 691 | 786 | 975 | 1047 | 354 | 1386 | 297 | 1839 | 522 | 684 | 168 | 1141 |
| *Coris cuvieri* | 1554 | 691 | 786 | 975 | 1047 | 354 | 1381 | 297 | 1842 | 522 | 684 | 168 | 1141 |
| *Coris julis* | 1551 | 691 | 786 | 975 | 1047 | 354 | 1380 | 297 | 1842 | 522 | 684 | 168 | 1141 |
| *Coris formosa* | 1554 | 691 | 786 | 975 | 1047 | 354 | 1381 | 297 | 1842 | 522 | 684 | 168 | 1141 |
| *Bodianus flavipinnis* | 1551 | 691 | 786 | 975 | 1047 | 351 | 1381 | 297 | 1839 | 522 | 684 | 168 | 1141 |
| *Halichoeres salmofasciatus* | 1551 | 691 | 786 | 975 | 1047 | 354 | 1377 | 297 | 1839 | 522 | 684 | 168 | 1141 |
| *Halichoeres caudalis* | 1551 | 691 | 786 | 975 | 1047 | 352 | 1380 | 297 | 1839 | 522 | 684 | 168 | 1141 |
| *Halichoeres adustus* | 1551 | 691 | 786 | 975 | 1047 | 354 | 1380 | 297 | 1839 | 522 | 684 | 168 | 1141 |
| *Halichoeres brasiliensis* | 1551 | 691 | 786 | 975 | 1047 | 354 | 1380 | 297 | 1839 | 522 | 684 | 168 | 1141 |
| *Choerodon oligacanthus* | 1551 | 691 | 786 | 975 | 1047 | 351 | 1381 | 297 | 1839 | 522 | 684 | 168 | 1141 |

| *Choerodon cephalotes* | 1551 | 691 | 786 | 975 | 1047 | 351 | 1381 | 297 | 1839 | 522 | 684 | 168 | 1141 |
| --- | --- | --- | --- | --- | --- | --- | --- | --- | --- | --- | --- | --- | --- |
| *Pseudodax moluccanus* | 1551 | 691 | 786 | 975 | 1047 | 351 | 1386 | 297 | 1839 | 522 | 684 | 168 | 1141 |
| *Coris picta* | 1551 | 691 | 786 | 975 | 1045 | 354 | 1381 | 297 | 1842 | 522 | 684 | 168 | 1141 |

*Supplementary Table 5a –* Average lengths of individual PCGs by genera (to the nearest bp) for all novel complete mitogenomes generated in this study

| **Genus** | **COI** | **COII** | **COIII** | **ND1** | **ND2** | **ND3** | **ND4** | **ND4L** | **ND5** | **ND6** | **ATP6** | **ATP8** | **CYTB** |
| --- | --- | --- | --- | --- | --- | --- | --- | --- | --- | --- | --- | --- | --- |
| *Bodianus* | 1551 | 691 | 786 | 975 | 1047 | 351 | 1381 | 297 | 1839 | 522 | 684 | 168 | 1141 |
| *Chlorurus* | 1551 | 691 | 786 | 972 | 1047 | 354 | 1380 | 297 | 1839 | 522 | 684 | 168 | 1141 |
| *Choerodon* | 1551 | 691 | 786 | 975 | 1048 | 351 | 1381 | 297 | 1839 | 522 | 684 | 168 | 1141 |
| *Cirrhilabrus* | 1554 | 691 | 786 | 975 | 1047 | 354 | 1381 | 297 | 1839 | 522 | 684 | 168 | 1141 |
| *Coris* | 1553 | 691 | 786 | 975 | 1047 | 354 | 1381 | 297 | 1841 | 522 | 684 | 168 | 1141 |
| *Epibulus* | 1551 | 691 | 786 | 975 | 1047 | 352 | 1386 | 297 | 1839 | 522 | 684 | 168 | 1141 |
| *Gomphosus* | 1551 | 691 | 786 | 975 | 1047 | 354 | 1380 | 297 | 1839 | 522 | 684 | 168 | 1141 |
| *Halichoeres* | 1557 | 691 | 786 | 975 | 1047 | 354 | 1380 | 297 | 1838 | 525 | 684 | 168 | 1141 |
| *Labroides* | 1551 | 691 | 786 | 975 | 1047 | 354 | 1386 | 297 | 1839 | 522 | 684 | 168 | 1141 |
| *Pseudodax* | 1551 | 691 | 786 | 975 | 1047 | 351 | 1386 | 297 | 1839 | 522 | 684 | 168 | 1141 |
| *Scarus* | 1551 | 691 | 786 | 973 | 1047 | 354 | 1381 | 297 | 1839 | 522 | 684 | 168 | 1141 |
| *Sparisoma* | 1551 | 691 | 786 | 975 | 1047 | 354 | 1381 | 297 | 1848 | 522 | 682 | 168 | 1141 |
| *Suezichthys* | 1551 | 691 | 786 | 975 | 1047 | 354 | 1386 | 297 | 1839 | 522 | 684 | 168 | 1141 |

*Supplementary Table 5b –* Average lengths of individual PCGs by tribe (to the nearest bp) for all novel complete mitogenomes generated in this study

| **Tribe** | **COI** | **COII** | **COIII** | **ND1** | **ND2** | **ND3** | **ND4** | **ND4L** | **ND5** | **ND6** | **ATP6** | **ATP8** | **CYTB** |
| --- | --- | --- | --- | --- | --- | --- | --- | --- | --- | --- | --- | --- | --- |
| Cheilininae | 1551 | 691 | 786 | 975 | 1047 | 352 | 1386 | 297 | 1839 | 522 | 684 | 168 | 1141 |
| *Cirrhilabrinae* | 1554 | 691 | 786 | 975 | 1047 | 354 | 1381 | 297 | 1839 | 522 | 684 | 168 | 1141 |
| *Hypsigenyinae* | 1551 | 691 | 786 | 975 | 1047 | 351 | 1382 | 297 | 1839 | 522 | 684 | 168 | 1141 |
| *Julidinae* | 1555 | 691 | 786 | 975 | 1047 | 354 | 1380 | 297 | 1839 | 524 | 684 | 168 | 1141 |
| Pseudolabrinae | 1551 | 691 | 786 | 975 | 1047 | 354 | 1386 | 297 | 1839 | 522 | 684 | 168 | 1141 |
| Scarinae | 1551 | 691 | 786 | 973 | 1047 | 354 | 1380 | 297 | 1841 | 522 | 684 | 168 | 1141 |





**SUPPLEMENTARY FIGURE 1** The total count of the 13 mitochondrial PCGs (blue) and two rRNAs (green) recovered from 388 UCE target

capture assemblies in Labridae.
